# The Role of Regional Anesthesia in Preventing Cancer Recurrence

**DOI:** 10.7759/cureus.97114

**Published:** 2025-11-17

**Authors:** Mark Salib, John Salib, David K Sum

**Affiliations:** 1 Medicine, St. George's University School of Medicine, St. George's, GRD; 2 Anesthesiology, Community First Medical Center, Chicago, USA

**Keywords:** anesthesia and cancer survival, anesthetic techniques, cancer recurrence, oncologic outcomes, perioperative analgesia, postoperative immunity, recurrence prevention, regional anesthesia, surgical stress response, tumor metastasis

## Abstract

Surgical resection remains the main curative approach for many solid cancers; however, recurrence and metastasis continue to present major challenges. Anesthetic techniques during the perioperative period may influence tumor behavior through their effects on immune and stress pathways. Regional anesthesia (RA) has been proposed as a potential strategy to reduce postoperative recurrence by limiting neuroendocrine activation, decreasing opioid use, and possibly exerting direct antitumor effects. A literature review was conducted to examine studies evaluating the relationship between RA and oncologic outcomes. Screening and synthesis were performed independently by multiple reviewers. Included studies encompassed laboratory, retrospective, prospective, and randomized research exploring both biological mechanisms and clinical outcomes. Experimental findings suggest that RA may help preserve immune function, reduce inflammation, and inhibit tumor growth and angiogenesis. Early clinical studies indicated possible benefits in recurrence-free survival, though more recent large-scale research has not confirmed a clear reduction in recurrence or mortality. Overall, evidence remains inconsistent and influenced by heterogeneity across study designs and cancer types. Despite uncertain oncologic effects, RA continues to offer well-established perioperative benefits, including improved pain control, reduced opioid use, and faster recovery. Further large, tumor-specific, and mechanism-focused trials are needed to clarify any potential long-term impact on cancer outcomes.

## Introduction and background

Cancer remains one of the leading causes of morbidity and mortality worldwide, with surgical resection serving as the cornerstone of curative therapy for most solid tumors [1]. Despite advances in surgical techniques and adjuvant therapies, recurrence and metastatic progression continue to represent noteworthy clinical challenges, significantly affecting long-term survival outcomes [2]. Increasing attention has been directed toward perioperative factors that may influence the trajectory of cancer recurrence. Among these, anesthetic techniques have emerged as a potential modifiable factor with biologic plausibility for impacting tumor progression [3].

The perioperative period is characterized by profound physiological stress, marked by the activation of the hypothalamic-pituitary-adrenal (HPA) axis, the release of catecholamines and inflammatory cytokines, and the suppression of cellular immune function [4]. In particular, natural killer (NK) cell activity, critical for surveillance against circulating tumor cells, is diminished during and after surgery [5]. In addition, opioids and volatile anesthetics, commonly used in perioperative care, may further exacerbate immunosuppression and promote pro-tumor pathways through various molecular mechanisms [6,7].

Regional anesthesia (RA) encompasses a broad range of techniques, including neuraxial methods such as spinal, epidural, and combined spinal-epidural blocks, as well as peripheral nerve blocks that target specific plexuses or individual nerves [3]. These modalities provide site-specific analgesia by intercepting afferent nociceptive transmission through the inhibition of voltage-gated sodium channels in neuronal membranes [4]. Beyond their analgesic benefit, RA procedures have been shown to modulate the perioperative stress response by attenuating sympathetic activation, reducing circulating catecholamines, and blunting the HPA axis response to surgical trauma [3,6]. Furthermore, laboratory data suggest that local anesthetics may exert direct antitumor effects, such as inhibition of cancer cell proliferation, migration, and angiogenesis [8]. Collectively, these mechanisms highlight the multifaceted role of RA in influencing not only perioperative outcomes but also long-term oncologic trajectories.

Over the past two decades, a growing body of preclinical studies, retrospective clinical analyses, and prospective trials has sought to evaluate the role of RA in cancer recurrence prevention. However, findings have been heterogeneous and sometimes conflicting [2,7]. This review aims to critically synthesize current evidence regarding the biological rationale, preclinical data, and clinical outcomes related to the use of RA in oncologic surgery, highlighting areas of consensus, ongoing controversy, and directions for future research.

## Review

Methods

**Figure 1:**
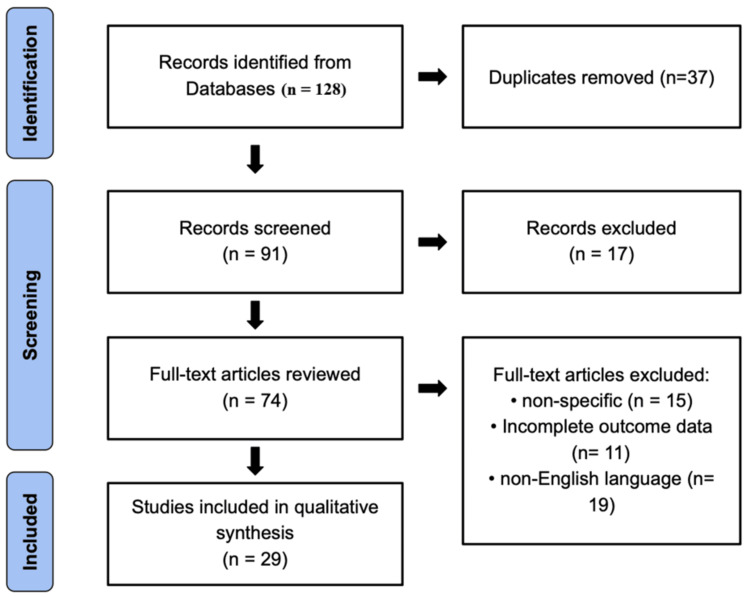
PRISMA flow diagram of study selection on the role of regional anesthesia in cancer recurrence prevention Flowchart illustrating the systematic identification, screening, eligibility, and inclusion process for studies evaluating surgical drain removal. A total of 128 records were identified through database searching, with 37 duplicates removed. After screening 91 records, 17 were excluded based on titles and abstracts. Of the 74 full-text articles reviewed, 45 were excluded for reasons including nonspecific focus (n = 15), incomplete outcome data (n = 11), and non-English language (n = 19). Ultimately, 29 studies were included in the qualitative synthesis PRISMA: Preferred Reporting Items for Systematic Reviews and Meta-Analyses

This review was conducted in accordance with the Preferred Reporting Items for Systematic Reviews and Meta-Analyses 2020 guidelines (as depicted in Figure 1) to ensure methodological rigor and transparency. A comprehensive literature search was performed across PubMed, MEDLINE, Embase, and Google Scholar for studies published in English between January 2010 and September 2025 that investigated the relationship between RA and cancer recurrence.

The search strategy incorporated both Medical Subject Headings and free-text keywords, including but not limited to RA, neuraxial anesthesia, epidural anesthesia, spinal anesthesia, paravertebral block, peripheral nerve block, local anesthetics, lidocaine, bupivacaine, ropivacaine, cancer recurrence, tumor metastasis, tumor progression, oncologic outcomes, immune modulation, immunosuppression, NK cells, neuroendocrine stress response, HPA axis, sympathetic activation, angiogenesis, inflammatory cytokines, opioid-sparing anesthesia, volatile anesthetics, propofol anesthesia, perioperative immunology, tumor microenvironment, and perioperative stress. Reference lists from relevant systematic reviews, meta-analyses, and landmark trials were manually screened to identify additional studies.

Three independent reviewers (blinded to each other’s findings) conducted the literature screening process in three stages: title screening, abstract screening, and full-text review. The collected literature (as depicted in Table 1) was appraised qualitatively and synthesized narratively. Studies were included if they evaluated the biological rationale, preclinical mechanisms, or clinical outcomes related to the impact of RA on cancer recurrence or survival. Eligible studies encompassed animal models, retrospective and prospective clinical analyses, randomized controlled trials (RCTs), and systematic or narrative reviews. Discrepancies were resolved by consensus discussion.

**Table 1:** Summary of studies evaluating RA and cancer recurrence This table summarizes key clinical and preclinical studies assessing the association between RA techniques and cancer recurrence or survival. It includes study design, cancer type, anesthetic approach, and main findings, highlighting the evolution of evidence from early retrospective reports to recent randomized trials and systematic reviews [1-29] GA: general anesthesia; RA: regional anesthesia; NK: natural killer; VEGF: vascular endothelial growth factor; IL-6: interleukin-6; PI3K: phosphoinositide 3-kinase; AKT: protein kinase B; MAPK: mitogen-activated protein kinase; TIVA: total intravenous anesthesia; TNF-α: tumor necrosis factor-alpha; DAMPs: damage-associated molecular patterns; HR: hazard ratio

Study	Study design/population	Surgical context/cancer type	Anesthetic/RA technique and comparator	Main findings
Le-Wendling et al. [1]	Narrative review	Various cancer surgeries	RA vs. GA	Comprehensive synthesis of immunologic and neuroendocrine pathways linking anesthesia to tumor recurrence. RA theorized to attenuate surgical stress, reduce catecholamine and opioid requirements, and preserve NK cell function, but large-scale evidence remains inconclusive
Boudreau et al. [2]	Retrospective cohort (n ≈ 6,000)	Breast cancer survivors	Chronic opioid use vs. no opioids	Long-term opioid therapy after initial treatment was associated with higher rates of second breast cancer events, suggesting opioid-induced immunosuppression may facilitate recurrence
Yeager and Rosenkranz [3]	Commentary	Multiple malignancies	RA vs. GA	Highlights emerging data that anesthetic choice could influence cancer outcomes. Advocates mechanistic research into immune preservation and stress modulation through RA
Bajwa et al. [4]	Narrative review	Multiple cancer types	RA vs. GA	Summarizes molecular and clinical data linking volatile anesthetics and opioids to tumor progression, while RA may limit recurrence by reducing perioperative stress and inflammation
Tedore [5]	Systematic review	Various cancers	RA vs. GA	Evidence supports possible oncologic benefits of RA, especially epidural and paravertebral blocks through reduced opioid exposure and improved immune competence, though results are inconsistent across trials
Vaghari et al. [6]	Review	Oncologic surgeries	RA with/without GA	Emphasizes dual benefit of RA: decreased infection rates and potential reduction in metastasis via sympathetic blockade and attenuated cortisol response
Cassinello et al. [7]	Review	Cancer surgery	RA and multimodal analgesia	Discusses perioperative immune suppression and the role of anesthetic choice; suggests RA may preserve cellular immunity and decrease tumor-promoting factors such as VEGF and IL-6
Singleton and Moss [8]	Hypothesis paper	Various	Opioid-based analgesia	Proposes opioids may enhance tumor growth by promoting angiogenesis and suppressing NK cell activity; urges minimizing perioperative opioid use when possible
Hiller et al. [9]	Review	Tumor resection	Various anesthetic strategies	Anesthetic drugs, opioids, and surgery-induced inflammation interact to influence tumor growth; highlights perioperative period as critical window for modulating recurrence risk
Piegeler et al. [10]	Experimental study (murine and in vitro)	Breast and lung cancer models	Lidocaine, ropivacaine	Demonstrated that amide-linked local anesthetics inhibit cancer cell migration, adhesion, and metastasis via Src signaling suppression, suggesting intrinsic antimetastatic potential
Kujawa et al. [11]	Narrative review	Breast cancer	RA vs. GA	Reviews evidence showing that paravertebral and epidural anesthesia reduce postoperative pain and inflammatory cytokine release, potentially improving recurrence-free survival
Wu et al. [12]	Review	Breast cancer	Various anesthetics	Anesthetics influence cancer cell signaling pathways (e.g., PI3K/AKT, MAPK). Suggests propofol and lidocaine exhibit antitumor properties, whereas volatile agents and opioids may promote tumor progression
Jin and Lew [13]	Review	Various oncologic surgeries	TIVA vs. volatile anesthesia	TIVA with propofol may reduce inflammatory responses, enhance cognitive recovery, and possibly lower cancer recurrence compared to volatile agents. Calls for prospective trials
Shin et al. [14]	Retrospective analysis (n = 3,000+)	Gastric cancer surgery	Epidural analgesia vs. systemic opioids	Patients receiving epidural analgesia had significantly higher 5-year overall survival and lower recurrence rates, suggesting beneficial immunomodulatory effects
Cong et al. [15]	Prospective cohort (n = 120)	Esophageal cancer	Epidural + GA vs. GA alone	RA-preserved postoperative NK and T-cell function, reduced IL-6 levels, and correlated with improved long-term survival
Ma et al. [16]	Retrospective observational (n = 400)	Esophageal cancer	Intravenous (propofol) vs. inhalational (sevoflurane) anesthesia	Propofol-based anesthesia associated with longer recurrence-free survival and fewer distant metastases than volatile anesthesia
Tseng et al. [17]	Review	Multiple malignancies	Various anesthetic types	Explores TNF-α modulation by anesthetics; highlights volatile agents’ potential to upregulate inflammatory cytokines, whereas propofol and lidocaine appear anti-inflammatory
Tang et al. [18]	Review	Surgical trauma and immune suppression	Perioperative immunology	Summarizes mechanisms of surgical stress-induced immunosuppression (e.g., catecholamines, glucocorticoids) and discusses RA as a potential countermeasure
Horner et al. [19]	Review	Surgical trauma	Immunologic effects of DAMPs	DAMPs suppress innate immunity and promote tumor seeding post-surgery; minimizing surgical stress and immune suppression (e.g., via RA) may reduce recurrence risk
Zhang et al. [20]	Systematic review and meta-analysis (16 studies)	Late-stage cancer surgeries	RA vs. GA	Found no significant overall effect of RA on recurrence or survival, though subgroup analyses suggested benefits in specific tumor types
Wang et al. [21]	Meta-analysis (eight cohort studies, n = 1,500+)	Nonmuscle invasive bladder cancer	RA vs. GA	RA associated with significantly lower recurrence rates; authors attribute effect to reduced immunosuppression and better perioperative oxygenation
Grandhi et al. [22]	Meta-analysis (20 studies)	Various cancers	RA vs. GA	Concluded that RA may improve overall and disease-free survival; emphasizes need for well-controlled prospective data
Xu et al. [23]	Randomized controlled trial (n = 400)	Lung cancer surgery	Epidural anesthesia-analgesia vs. GA	No difference in recurrence-free survival at 3 years; validates RA as safe without negative oncologic impact
Sekandarzad et al. [24]	Review	Oncologic surgery	RA and multimodal analgesia	Supports RA for its opioid-sparing and immune-preserving effects, and encourages integration into enhanced recovery protocols
Bhuyan et al. [25]	Narrative review	Various cancer surgeries	Optimized RA techniques	Summarizes practical RA approaches for oncology patients, emphasizing timing, multimodal integration, and immune protection
Lu et al. [26]	Systematic review and meta-analysis (21 studies)	Abdominal cancer	RA vs. GA	Found RA improved overall survival (HR = 0.86) and decreased recurrence risk, especially in gastrointestinal and urologic cancers
Wall et al. [27]	Narrative review	Cancer surgery	Various anesthetic and analgesic interventions	Highlights multifactorial influences on tumor recurrence, including anesthetic choice, opioid dose, and perioperative stress
Hiller et al. [28]	Narrative review (nature reviews)	Various cancers	Perioperative factors	Identifies inflammation, immune dysregulation, and angiogenesis during surgery as key drivers of recurrence; anesthesia plays a modifiable role
Saran et al. [29]	Retrospective study (n = 120)	Breast tumor resection	Single-injection vs. multilevel paravertebral block	Both techniques provided equivalent analgesia; ultrasound-guided single-injection was faster, safer, and equally effective for tumor resection

Data extraction was performed using a standardized template capturing study design, population characteristics, cancer type, anesthetic technique, comparator group, duration of follow-up, and reported oncologic outcomes (e.g., recurrence-free survival, overall survival, and biochemical recurrence). Due to significant heterogeneity in cancer types, anesthetic protocols, and outcome measures, a qualitative narrative synthesis was employed rather than quantitative meta-analysis. Findings were summarized in tabular and descriptive form to identify trends, mechanistic insights, and gaps in the current literature.

Biological rationale

The hypothesis that RA may reduce cancer recurrence is rooted in understanding the perioperative stress response and its effects on host immunity. Surgical trauma initiates activation of the sympathetic nervous system and the HPA axis, releasing catecholamines and cortisol [4]. These mediators suppress cellular immune function, impair NK cell cytotoxicity, and enhance angiogenesis, creating a favorable environment for residual tumor cells to proliferate and metastasize [5,9].

Opioids, commonly administered for perioperative analgesia, may further exacerbate immunosuppression. Experimental studies suggest that μ-opioid receptor activation can impair NK cell activity and promote tumor angiogenesis through vascular endothelial growth factor upregulation [6,10]. In addition, opioids have been shown to directly enhance cancer cell proliferation and migration in vitro, raising concern that opioid exposure during the perioperative period may accelerate tumor progression [10].

Volatile anesthetics may also play a role. In vitro studies have demonstrated that halogenated anesthetics can upregulate hypoxia-inducible factors, alter gene expression, and suppress cell-mediated immunity [11]. Conversely, propofol, commonly used in total intravenous anesthesia, preserves NK cell activity and exerts anti-inflammatory and antioxidant effects [12].

RA offers a mechanism to attenuate these detrimental pathways. By providing superior analgesia and reducing or eliminating the need for perioperative opioids, regional techniques may decrease opioid-related immunosuppression [3,6]. Moreover, RA blunts the neuroendocrine stress response, reducing catecholamine and cortisol release and preserving immune surveillance [4,9].

Local anesthetics themselves may have direct anticancer properties. Laboratory studies have shown that agents such as lidocaine and ropivacaine can inhibit tumor cell proliferation, migration, and invasion, and may even induce apoptosis in distinctive cancer cell lines [8,13]. These findings suggest that regional techniques not only modulate the host response but may also act directly on tumor biology.

Collectively, these mechanisms (as depicted in Table 2) provide a biologically plausible framework through which RA could reduce the risk of postoperative cancer recurrence and metastasis. However, translating these effects from preclinical and mechanistic studies into clinical benefit remains a subject of ongoing investigation.

**Table 2:** Proposed mechanisms by which RA may influence cancer recurrence RA may attenuate the perioperative stress response, reduce reliance on opioids and volatile anesthetics, and deliver local anesthetics with potential direct antitumor effects. Collectively, these mechanisms provide a biologically plausible framework linking anesthetic technique to oncologic outcomes [3-6,8-13] RA: regional anesthesia; SNS: sympathetic nervous system; HPA: hypothalamic-pituitary-adrenal; NK: natural killer; VEGF: vascular endothelial growth factor; TIVA: total intravenous anesthesia

Factor	Effect on cancer biology/immunity	Impact of RA
Surgical stress response(SNS + HPA axis activation → catecholamines, cortisol)	Suppresses cellular immunity, impairs NK cell cytotoxicity, and promotes angiogenesis, creating a favorable environment for tumor growth and metastasis [4,5,9]	RA blunts the neuroendocrine stress response, reduces catecholamine and cortisol release, and preserves immune surveillance [4,9]
Opioids	μ-opioid receptor activation impairs NK cells, promotes angiogenesis via VEGF, and enhances tumor proliferation and migration in vitro [6,10]	RA reduces opioid requirements, decreasing opioid-related immunosuppression and tumor-promoting effects [3,6,10]
Volatile anesthetics	Halogenated agents upregulate hypoxia-inducible factors, alter gene expression, and suppress cell-mediated immunity [11]	RA reduces or avoids volatile use; propofol-based TIVA preserves NK cell activity and exerts anti-inflammatory and antioxidant effects [12]
Local anesthetics (e.g., lidocaine, ropivacaine)	Demonstrated to inhibit tumor proliferation, migration, and invasion and may induce apoptosis in certain cancer cell lines [8,13]	RA delivers local anesthetics that may exert direct anticancer effects [8,13]

Preclinical and mechanistic evidence

A substantial and growing body of preclinical research supports the hypothesis that RA may attenuate cancer progression through preservation of host immunity and direct antitumor effects. Foundational animal studies demonstrated that surgical stress profoundly suppresses NK cell activity, a critical component of early tumor immune surveillance, accelerating metastatic dissemination. In contrast, adrenergic and prostaglandin signaling attenuation has been shown to restore NK cell cytotoxicity, limit metastatic colonization, and reduce overall tumor burden [9,14]. These mechanistic insights established a compelling biologic rationale for investigating whether anesthetic management in the perioperative period can modulate similar pathways in patients with cancer.

Rodent models have since provided more robust and direct support for this concept. Jin and Lew reported that perioperative sympathetic activation significantly increased pulmonary tumor retention. In contrast, pharmacologic or anesthetic interventions that blunted the stress response preserved NK cell function and sharply reduced metastatic spread [13]. Additional experimental work has demonstrated survival benefits when RA was combined with systemic β-blockade, suggesting a synergistic effect in mitigating perioperative neuroendocrine-driven tumor progression [14]. These findings reinforce the hypothesis that interventions targeting both surgical stress and immune modulation may alter the trajectory of cancer recurrence.

Beyond effects on host immunity, local anesthetics themselves appear to exert tumor-suppressive properties. In vitro studies consistently show that lidocaine, ropivacaine, and bupivacaine inhibit tumor cell proliferation, migration, and invasion across diverse malignancies, including breast, thyroid, and prostate cancer [13,15]. Mechanistic analyses indicate that these agents promote caspase-mediated apoptosis, disrupt mitochondrial integrity, and interfere with voltage-gated sodium channel signaling, a pathway increasingly implicated in tumor invasiveness and metastatic potential [15,16]. Such findings suggest that RA may exert systemic benefits through stress attenuation and direct cellular effects on malignant tissue.

RA techniques may further modulate the perioperative tumor microenvironment by reducing angiogenesis and inflammation. Laboratory evidence has linked exposure to local anesthetics with decreased vascular endothelial growth factor expression, reduced release of proinflammatory cytokines such as interleukin-6, and diminished activity of tumor-associated macrophages [10,17]. By suppressing these proangiogenic and immunosuppressive signals, RA may help prevent the establishment of a microenvironment conducive to micrometastatic growth and long-term disease progression.

While these preclinical findings are biologically persuasive and provide a coherent mechanistic framework, translation into consistent clinical benefit has remained elusive. Variability in tumor biology, differences in anesthetic protocols, and heterogeneity in host immune responses complicate the extrapolation of laboratory data to clinical outcomes. Nevertheless, preclinical research continues to deliver crucial mechanistic insights, underscoring the plausibility that anesthetic technique can influence cancer biology and justifying the need for rigorously designed clinical trials with tumor-specific endpoints and long-term follow-up.

Clinical evidence

The potential influence of RA on long-term oncologic outcomes has been the subject of extensive clinical investigation. Initial evidence arose from retrospective analyses that generated considerable enthusiasm. Yeager and Rosenkranz discussed the potential influence of anesthetic technique on cancer recurrence, emphasizing that RA may attenuate perioperative immunosuppression and thereby reduce the likelihood of tumor dissemination and metastatic progression [3]. Similarly, Biki et al. observed lower rates of biochemical recurrence among patients receiving epidural anesthesia and analgesia during radical prostatectomy, compared with those managed exclusively with general anesthesia (GA) and opioids [6]. These early findings provided the rationale for prospective evaluation.

However, subsequent retrospective studies produced mixed results. Several analyses failed to demonstrate an association between RA and improved recurrence-free or overall survival, raising concerns that earlier observations may have been confounded by factors such as surgical complexity, baseline comorbidities, or institutional variation in anesthetic practice [18,19].

Prospective RCTs were undertaken to address these limitations. The largest trial to date, conducted by Cassinello et al., randomized more than 2,000 women undergoing curative breast cancer surgery to either paravertebral block with propofol anesthesia or GA with sevoflurane and opioids. After nearly five years of follow-up, no differences were observed in breast cancer recurrence or overall survival [7]. Additional smaller RCTs in colorectal and abdominal cancer surgery similarly failed to demonstrate oncologic benefit from RA [20].

Evidence synthesis through meta-analysis has likewise produced inconsistent conclusions. Some systematic reviews suggested a modest protective effect of RA in selected tumor types. In contrast, others concluded that heterogeneity in study design and the predominance of retrospective data preclude firm conclusions [21,22]. These findings highlight the inherent challenges of studying long-term cancer outcomes, where perioperative management, tumor biology, and host factors converge.

Taken together, current clinical evidence does not provide definitive support for RA as a strategy to reduce cancer recurrence. Nonetheless, RA retains a central role in perioperative care due to its established benefits in analgesia, opioid reduction, and enhanced recovery. At the same time, its potential oncologic effects remain an active and important area of investigation.

Current consensus

Despite compelling mechanistic data and promising results from select retrospective studies, consensus in the clinical community regarding RA and cancer recurrence has not been achieved (as depicted in Table 3). Recent comprehensive narrative reviews and meta-analyses consistently conclude that high-quality evidence is insufficient to establish a causal relationship between regional techniques and long-term oncologic outcomes [23,24]. The largest RCT to date, which compared paravertebral block plus propofol with volatile anesthesia and opioids in women undergoing breast cancer surgery, demonstrated no difference in recurrence rates, tempering the early enthusiasm generated by retrospective reports [7,25]. Additional RCTs and pooled analyses in colorectal and other abdominal malignancies have similarly failed to demonstrate a consistent oncologic benefit attributable to RA [20,26].

**Table 3:** Clinical evidence evaluating the impact of RA on cancer recurrence This table summarizes key clinical studies evaluating the impact of RA on long-term oncologic outcomes. Early retrospective studies suggested potential reductions in cancer recurrence in breast and prostate cancer [3,6], whereas later retrospective analyses produced mixed results [18,19]. Large prospective RCTs, including Cassinello et al., demonstrated no difference in recurrence or overall survival [7,20,25,26], and smaller RCTs in colorectal and abdominal cancers yielded similar findings [20,26]. Current professional guidelines do not recommend RA solely for cancer recurrence prevention, highlighting established benefits in perioperative analgesia, opioid reduction, and enhanced recovery [20-29] RA: regional anesthesia; GA: general anesthesia; RCT: randomized controlled trial

Evidence level/study type	Key studies	Patient population/cancer type	RA intervention	Comparator	Key findings/outcomes
Early retrospective	[21,29]	Breast cancer; prostate cancer	Paravertebral block; epidural anesthesia	GA + opioids	Suggested reduced cancer recurrence and biochemical recurrence with RA
Later retrospective	[24,27,28]	Various solid tumors	Epidural or paravertebral block	GA + opioids	Mixed findings; no consistent association with improved recurrence-free or overall survival
Prospective RCT (large)	[23,25]	Breast cancer (n > 2,000)	Paravertebral block + propofol	GA + sevoflurane + opioids	No difference in cancer recurrence or overall survival
Prospective RCT (smaller)	[20,26]	Colorectal, abdominal cancers	RA	GA + opioids	No consistent oncologic benefit observed
Guideline Recommendations	[22,27,28]	N/A	N/A	N/A	RA not recommended solely for cancer recurrence prevention; use guided by analgesic, opioid-sparing, and recovery benefits

Accordingly, professional societies do not recommend the routine use of RA solely for cancer recurrence prevention. Guidelines instead emphasize well-established indications for RA, including superior perioperative analgesia, opioid-sparing effects, reduced pulmonary complications in selected populations, and facilitation of enhanced recovery protocols [22,27]. The inability to make a definitive recommendation reflects multiple factors: tumor heterogeneity, variation in anesthetic and surgical protocols, differences in adjuvant therapy, and the predominance of retrospective data susceptible to confounding [23,26,28].

Clinicians are advised to individualize anesthetic management, prioritizing multimodal strategies that optimize short-term outcomes and recovery, while recognizing that any potential long-term oncologic benefits of RA remain unproven. Guideline committees and research bodies continue to advocate for rigorously designed randomized trials and pooled prospective data collection to address these uncertainties and clarify the role of RA in oncologic outcomes [23,27].

Future directions

The limitations of existing evidence highlight clear priorities for future research. Large, adequately powered RCTs with standardized anesthetic protocols and long-term follow-up are essential. Such trials should prespecify oncologic endpoints, including recurrence-free and overall survival, stratify by tumor type and stage, and rigorously document perioperative opioid use and adjuvant therapies [23,26]. Tumor-specific investigations are likely to be more informative than heterogeneous cohorts, as mechanistic rationale and retrospective signals suggest that breast, colorectal, and prostate cancers may warrant prioritization [3,20,21].

Mechanistic and translational studies should be embedded within clinical trials, with perioperative biomarker panels assessing NK cell activity, circulating tumor cells, cytokine profiles, and angiogenesis markers to link anesthetic interventions to biologic effects and identify patient subgroups most likely to benefit [5,9,29]. Multimodal perioperative strategies that combine RA with immune-preserving interventions such as total intravenous anesthesia with propofol, β-blockade, cyclooxygenase-2 inhibition, or perioperative immunotherapy merit exploration in factorial trial designs [12,14,21].

Prospective consortia and registries that collect standardized perioperative and oncologic outcome data will accelerate evidence generation and enable robust subgroup and interaction analyses [23]. Incorporating cost-effectiveness assessments and patient-centered outcomes is also critical, as even modest oncologic benefits must be weighed against practical considerations and established advantages in analgesia and recovery.

Overall, the field is evolving from early observational enthusiasm to a measured, evidence-driven approach. RA remains a key component of perioperative care for its analgesic and recovery benefits, and ongoing high-quality clinical trials with integrated translational science are essential to determine whether it can meaningfully influence cancer recurrence.

## Conclusions

RA continues to hold an important position in modern perioperative practice due to its proven benefits in analgesia, reduced opioid consumption, lower pulmonary and cardiovascular complications, and facilitation of enhanced recovery after surgery. The hypothesis that regional techniques may also confer long-term oncologic advantages remains biologically plausible and mechanistically supported by preclinical evidence. Studies in animal and cellular models demonstrate that attenuation of the perioperative stress response, preservation of NK cell function, and direct cytotoxic effects of local anesthetics could collectively limit tumor proliferation and metastasis. However, translating these mechanistic insights into consistent clinical benefit has been challenging. Retrospective studies initially suggested improved recurrence-free survival in certain malignancies. However, larger RCTs and meta-analyses have failed to confirm a statistically significant impact of regional anesthesia on recurrence or overall survival. This inconsistency likely reflects multifactorial influences on tumor biology, differences in anesthetic technique, tumor heterogeneity, and variability in adjuvant therapy and long-term follow-up.

The current body of evidence does not justify the use of regional anesthesia solely for the purpose of reducing cancer recurrence. Instead, it should continue to be utilized for its established perioperative advantages, within a multimodal framework that prioritizes optimal pain control, physiologic stability, and rapid recovery. Future research should bridge the gap between biologic plausibility and clinical translation through rigorously designed, tumor-specific randomized trials incorporating standardized anesthetic protocols, long-term oncologic follow-up, and mechanistic biomarker analyses. Ultimately, while regional anesthesia may yet prove to have secondary oncologic benefits in selected patient populations, its primary role remains that of an evidence-based component of comprehensive, patient-centered perioperative care.
